# The added value of spinal cord lesions to disability accrual in multiple sclerosis

**DOI:** 10.1007/s00415-023-11829-5

**Published:** 2023-06-29

**Authors:** Serena Ruggieri, Luca Prosperini, Maria Petracca, Alessandra Logoteta, Emanuele Tinelli, Laura De Giglio, Olga Ciccarelli, Claudio Gasperini, Carlo Pozzilli

**Affiliations:** 1 Department of Human Neurosciences, Sapienza University of Rome, Viale Dell’Università 30, 00185 Rome, Italy; 2 Neuroimmunology Unit, IRCSS Fondazione Santa Lucia, Rome, Rome, Italy; 3 Department of Neurosciences, San Camillo-Forlanini Hospital, Rome, Italy; 4 Department of Maternal Infantile and Urological Sciences, Sapienza University of Rome, Rome, Italy; 5 Unit of Neuroradiology, Department of Medical and Surgical Sciences, “Magna Graecia” University, Catanzaro, Italy; 6 Radiology, Neurological Center of Latium, Rome, Rome, Italy; 7 Neurology Unit, San Filippo Neri Hospital, Rome, Italy; 8 Queen Square MS Centre, Faculty of Brain Sciences, University College London Queen Square Institute of Neurology, London, UK; 9 National Institute for Health Research Biomedical Research Centre, University College London Hospitals, London, UK

**Keywords:** Spinal cord MRI, Disability accrual, Asymptomatic lesions, Treatment optimization

## Abstract

Spinal cord MRI is not routinely performed for multiple sclerosis (MS) monitoring. Here, we explored whether spinal cord MRI activity offers any added value over brain MRI activity for clinical outcomes prediction in MS. This is a retrospective, monocentric study including 830 MS patients who underwent longitudinal brain and spinal cord MRI [median follow-up 7 years (range: < 1–26)]. According to the presence (or absence) of MRI activity defined as at least one new T2 lesion and/or gadolinium (Gd) enhancing lesion, each scan was classified as: (i) brain MRI negative/spinal cord MRI negative; (ii) brain MRI positive/spinal cord MRI negative; (iii) brain MRI negative/spinal cord MRI positive; (iv) brain MRI positive/spinal cord MRI positive. The relationship between such patterns and clinical outcomes was explored by multivariable regression models. When compared with the presence of brain MRI activity alone: (i) Gd + lesions in the spine alone and both in the brain and in the spinal cord were associated with an increased risk of concomitant relapses (OR = 4.1, 95% CI 2.4–7.1, *p* < 0.001 and OR = 4.9, 95% CI 4.6–9.1, *p* < 0.001, respectively); (ii) new T2 lesions at both locations were associated with an increased risk of disability worsening (HR = 1.4, 95% CI = 1.0–2.1, *p* = 0.05). Beyond the presence of brain MRI activity, new spinal cord lesions are associated with increased risk of both relapses and disability worsening. In addition, 16.1% of patients presented asymptomatic, isolated spinal cord activity (Gd + lesions). Monitoring MS with spinal cord MRI may allow a more accurate risk stratification and treatment optimization.

## Introduction

Spinal cord is affected by both inflammatory and neurodegenerative changes in patients with multiple sclerosis (MS) [1]. Studies using magnetic resonance imaging (MRI) have demonstrated that the presence and the number of spinal cord lesions predict conversion to clinically definite MS in patients with a clinically isolated syndrome (CIS) [2] as well as short- [3] and long-term disability accrual in patients with established MS [4], independently of brain damage [5, 6]. In addition, a high risk of disability worsening is associated with new spinal cord lesions during the first year of treatment with interferons [7], supporting the recommendation to switch to high-efficacy treatments in patients with high spinal cord lesion load [8, 9]. However, the added value of a systematic MRI protocol, including spinal cord imaging in monitoring the disability accrual in MS, is unknown.

Only few MS centers adopt routine spinal cord MRI to monitor disease evolution and treatment response in patients with MS [10]. Given the challenges related to acquisition and interpretation of spinal cord images, as well as time and cost considerations, and because of the evidence that spinal cord MRI is of little value to monitor disease activity in MS, current recommendations on the use of MRI in patients with MS advise against the use of routine spinal cord MRI and recommend it only in a few special circumstances [11].

Thus, here, we aimed to: (1) establish the frequency of spinal cord inflammatory MRI activity occurring independently of brain activity; (2) investigate the association between MRI activity in the spinal cord, alone or in combination with brain activity and relapses; and (3) investigate the association between MRI activity in the spinal cord, alone or in combination with brain activity and disability accrual.

## Materials and methods

### Study design and participants

In this observational, retrospective study, we extracted MRI, demographic and clinical data from the MS Registry of the Multiple Sclerosis Center of St. Andrea Hospital in Rome (Italy), which includes 1335 patients, whose data have been prospectively collected by the treating clinicians at each visit (every 6 months or every year) since 2001. Of note, the registry, although established in 2001, also contains retrospective data collected in paper charts before 2001.

For each patient at each encounter, we collected the following demographic, clinical and MRI data: sex, date of birth, date of disease onset, date of diagnosis, disease course, disease-modifying treatment (DMT), symptomatic treatments, relapses, and Expanded Disability Status Scale (EDSS) [12], number of new T2-hyperintense lesions and gadolinium-enhancing (Gd +) lesions and their location, as described in the MRI report and re-assessed by the treating clinicians upon MRI images review. In our Center, spinal cord MRI scans are routinely performed together with brain MRI scan, as part of the patients’ follow-up.

The Ethical Committee board of Sapienza University of Rome at Sant’Andrea Hospital provided approval for the project. Informed, written consent was obtained by all patients.

Patients were enrolled when fulfilling the following inclusion criteria:

1. Diagnosis of MS according to the 2017 revised McDonald Criteria [13];
2. At least two MRI scans acquired with a minimal interval of 30 days, on the same 1.5 T scanner (GE Signa Excite) and with the same MRI protocol (details below), including both brain and spinal cord MRI (cervical and thoracic MRI) before and after Gd administration.
3. Neurological examination, which included the EDSS, within 30 days of the MRI scan (neurological assessments out of this time window were not considered).

Disability worsening was defined as an increase in the EDSS score of 1.5 points for patients with previous EDSS score of 0, 1 point for scores from 1.0 to 5.0, and 0.5 points for scores equal or higher to 5.5 [14]. Disability worsening was estimated over the time elapsed between each scan and the following scan throughout the entire study period.

A clinical relapse was defined as any new neurologic symptom not associated with fever or infection lasting for at least 24 h and accompanied by new neurologic signs [13].

### MRI acquisition and analysis

MRI scans were performed on the same 1.5 T scanner using eight-channel receive-only neurovascular head coil with a standard protocol in accordance with Italian Guidelines [15]. For brain study, MRI scans were performed with the following protocol: dual-echo proton density (PD)-T2-weighted images (repetition time (TR) = 2450 ms; echo time (TE) = 18/115 ms), with axial 4.0 mm thickness, gap 0.4 mm, matrix = 512 × 512, field of view (FOV) = 250 × 250 mm, and 64 interleaved slices; fast fluid-attenuated inversion recovery (FLAIR) (TR = 8002 ms, TE = 98 ms, inversion time (TI) = 2000 ms) with axial 4.0 mm thickness, gap 0.4 mm, matrix = 512 × 512, FOV = 250 × 250 mm, and 32 contiguous slices. Post-contrast T1-weighted spin-echo images after gadolinium diethylenetriamine penta-acetic acid (Gd-DTPA) double-dose injection (0.2 mmol/kg) (TR = 520 ms; TE = 21 ms; axial 4.0-mm-thick slices with axial 4.0 mm thickness, gap 0.4 mm, matrix = 512 × 512, FOV = 250 × 250 mm, 32 contiguous slices) were also obtained.

In the same scanning session, the following pulse sequences were used to acquire the cervical and thoracic spinal cord: sagittal T2-weighted spin echo with a slice-thickness of 3.0 mm (TR = 2800 ms; TE = 119 ms), with a gap between slices of 0.3 mm, matrix = 320 × 256; axial T2-fast spin echo with a slice-thickness of 4.0 mm (TR: 4154 ms, TE: 102 ms), matrix: 288 × 192; short tau inversion recovery (STIR) with a slice-thickness of 3.0 mm (TR = 2350 ms; TE = 46 ms; TI = 200 ms), with a gap between slices of 0.3 mm, matrix = 256 × 160; post-contrast-enhanced T1-weighted scans were acquired between 5 and 10 min after injection of 0.2 mmol/kg of Gd-DTPA (TR = 441 ms; TE = 11 ms), with a gap between slices of 0.3 mm, matrix = 320 × 256.

From 2017, in accordance with EMA recommendations [16], all post-contrast scans were acquired after injection of a 0.1 mmol/kg of gadoteric acid.

A neuroradiologist with an experience of more than 15 years (E.T.), blinded to the patients’ clinical data, reviewed all the scans at each time point and filled a report indicating the number of new brain and spinal cord T2 lesions as well Gd + lesions as per clinical practice in our center. A lesion was defined as new if it was not present on the previous MRI scan. Enlarging T2-hyperintense lesions were not scored due to the poor between-rater agreement in the clinical setting [17]. On the basis of the presence (or absence) of at least one new T2 lesion or one Gd + lesion, each MRI scan was classified into one of the following four patterns: (i) brain MRI negative/spinal cord MRI negative; (ii) brain MRI positive/spinal cord MRI negative; (iii) brain MRI negative/spinal cord MRI positive; (iv) brain MRI positive/spinal cord MRI positive. Asymptomatic lesions were defined as Gd + lesions on MRI scans of patients who did not show a relapse within 30 days prior to, or within 30 days after, the MRI scan.

### Statistical analysis

To address the aims of the study, each MRI scan was considered as a statistical unit. Categorical data were presented as count (proportion); continuous data were presented as median (range), or mean (standard deviation), as appropriate.

The relationship between MRI patterns and concomitant relapses (dependent variable) was explored by multivariable logistic regression models adjusted by sex, age, disease duration, EDSS score, and DMT. DMT was modeled as a three-level categorical variable, as follows: none (reference value), lower efficacy drug (i.e., interferon beta, glatiramer acetate, dimethyl fumarate, teriflunomide, azathioprine) and higher efficacy drug (i.e., natalizumab, mitoxantrone, and fingolimod).

The variable “MRI pattern” was modeled as a multilevel category (e.g., brain MRI negative/spinal cord MRI negative, brain MRI positive/spinal cord MRI negative, brain MRI negative/spinal cord MRI positive, brain MRI positive/spinal cord MRI positive). Between group post hoc comparisons were carried out versus the reference category (which was brain MRI negative/spinal cord MRI negative) and then in a repeated way versus the previous category. Results are expressed as Odds Ratio (OR) with 95% Confidence intervals (CI) and Nagelkerke pseudo-*R*^2^, which quantified the amount of variance for each model.

The relationship between MRI patterns and disability accrual (dependent variable) was explored by multivariable Cox regression models adjusted by sex, age, disease duration, EDSS score, DMT, and concomitant relapses. To explore the effect of asymptomatic spinal cord lesions on the risk of disability accrual we repeated the analysis using Gd + MRI scans without a concomitant relapse. The time elapsed between one scan and the next scan of each patient was set as main time variable; therefore, we excluded from the survival analysis the last available scans for all included patients. All the models were weighted for the inverse number of scans per patient, to avoid the overestimation of data from patients who had more scans. Lastly, to account for the correlation between data from the same patient in the scan-wise analyses, all models were stratified by individual patient.

*P*-value < 0.05 was considered statistically significant and were reported.

## Results

### Study population and overview of MRI data

Out of 1335 patients included in the MS Registry of St Andrea MS Centre, 830 patients were enrolled (Table 1). The remaining 505 patients were excluded because they had MRI exams performed on different MRI scanners over the follow-up, either because they were originally followed in other MS Centers or as a result of their personal choice to perform MRI exams elsewhere. At the time of the first scan, the majority of the cohort had relapsing remitting (RRMS) (87.8%), a mean disease duration of 13.6 years (SD = 9.7), and a median disability of 2 (range 0–7.5) (Table 1). A total of 5701 MRI exams were reviewed, with a median number of 6 (range 2–22) sessions per patient. The median length of follow-up (time between the first and the last available session) was 7 years (range < 1–26).

**Table 1:** Demographic and clinical characteristics of the whole cohort (*n* = 830 patients) at study entry

Sex, *n* (%)	
Female	574 (69.2%)
Male	256 (30.8%)
Age, years	
Mean (SD)	34.6 (9.7)
Median (range)	34.0 (10–68)
Disease duration, years	
Mean (SD)	13.6 (7.9)
Median (range)	12.0 (< 1–45)
Disease-modifying therapy, *n* (%)	
Yes	559 (67.3)
• Injectables	404 (48.7)
• Azathioprine	48 (5.8)
• Natalizumab	44 (5.3)
• Mitoxantrone	43 (5.2)
• Fingolimod	9 (1.1)
• Dimethyl fumarate	6 (0.7)
• Teriflunomide	5 (0.6)
No	271 (32.7)
Disease course, *n* (%)	
RRMS	729 (87.8%)
SPMS	45 (5.4%)
PPMS	56 (6.7%)
EDSS score	
Median (range)	2.0 (0–7.5)

Abbreviations: *EDSS* Expanded Disability Status Scale, *PPMS* primary progressive multiple sclerosis, *RRMS* relapsing remitting multiple sclerosis, *SPMS* secondary progressive multiple sclerosis

### Frequency and degree of brain and spinal cord inflammatory activity

#### Scan-wise

**Fig. 1:**
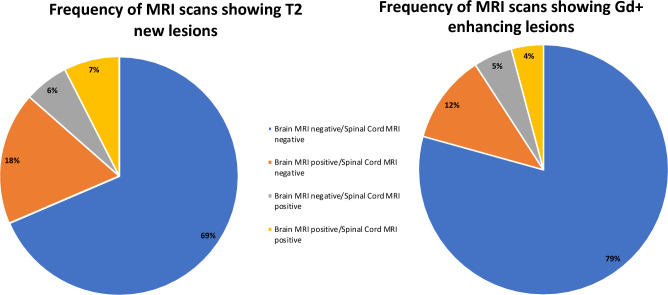
Frequency of MRI scans according to each pattern

Out of 5701 MRI exams, 1022 (17.9%) showed new T2 lesions located exclusively in the brain (brain MRI positive/spinal cord MRI negative), 342 (6%) showed new T2 lesions in the spinal cord alone (brain MRI negative/spinal cord MRI positive), and 428 (7.5%) showed a concomitant presence of brain and spinal cord new T2 lesions (brain MRI positive/spinal cord MRI positive); 3909 (68.6%) MRI scans did not show new T2 lesions in the brain or spinal cord (brain MRI negative/spinal cord MRI negative) (Fig. 1).

When Gd activity was explored, 657 (11.5%) MRI scans showed Gd + lesions in the brain alone (brain MRI positive/spinal cord MRI negative), 286 (5%) MRI scans showed Gd + lesions only in the spinal cord (brain MRI negative/spinal cord MRI positive), and 237 (4.2%) MRI scans showed Gd + lesions in both the brain and spinal cord (brain MRI positive/spinal cord MRI positive). The majority of the MRI scans did not show any Gd + lesions in the brain or spinal cord (brain MRI negative/Spinal cord negative) (*n* = 4521, 79.3%) (Fig. 1).

When looking at the number of new lesions in the brain, the majority of the scans showed no new T2 or Gd + lesions (74.6% and 84.3%, respectively), followed by the scans which showed one new T2 or Gd + lesion (11.4% and 8.1%, respectively) (Table 2). When investigating the number of new lesions in the spinal cord, the majority of the scans showed no new T2 or Gd + lesions (86.5% and 90.8%, respectively), followed by the scans which showed one new T2 or Gd + lesion (9.7% and 7.3%, respectively) (Table 2). Specifically, among all scans showing at least one new T2 lesions in the spinal cord, 439 (7.7%) showed lesions in the cervical spine, 215 (3.8%) in the thoracic spine and 116 (2.0%) in both. Among all scans showing new Gd + spinal cord lesions, 315 (5.5%) showed lesions in the cervical spine, 156 (2.7%) in the thoracic spine and 52 (0.9%) at both levels.

**Table 2:** Number of new T2 lesions and Gd + lesions in the brain and spinal cord (*n* = 5701)

	Number	New T2 lesions	Gd + lesions
Brain	0	4251 (74.6%)	4807 (84.3%)
	1	648 (11.4%)	463 (8.1%)
	2	331 (5.8%)	171 (3.0%)
	3	172 (3.0%)	97 (1.7%)
	4	110 (1.9%)	61 (1.1%)
	≥ 5	189 (3.3%)	102 (1.8%)
Spinal cord	0	4931 (86.5%)	5178 (90.8%)
	1	554 (9.7%)	419 (7.3%)
	2	141 (2.5%)	67 (1.2%)
	≥ 3	75 (1.3%)	37 (0.7%)

*In brackets the percentage of scans showing number of new T2 or Gd* + *lesions*

**Fig. 2:**
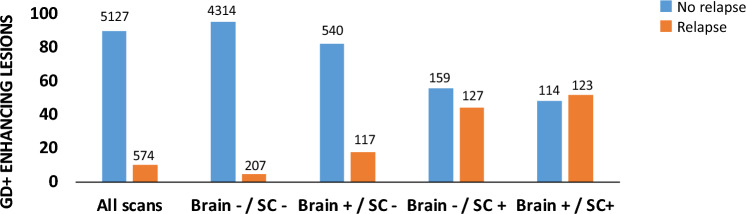
Associations of MRI scans with clinical relapses according to each pattern. The ordinal axis shows percentage of scan of the total for each pattern. The number on the top of each bar shows the absolute number of scans for each pattern. Abbreviations: *Gd + * Gadolinium enhancing lesions, *Brain–/SC–* brain MRI negative/spinal cord MRI negative, *Brain + /SC–* brain MRI positive/spinal cord MRI negative, *Brain–/SC + * brain MRI negative/spinal cord MRI positive, *Brain + /SC + * brain MRI positive/spinal cord MRI positive)

When examining asymptomatic Gd activity, 540 out of 5701 MRI scans (9.5%) showed asymptomatic Gd + lesions in the brain alone (brain MRI positive/spinal cord MRI negative), 159 (2.8%) MRI scans showed asymptomatic Gd + lesions in the spinal cord alone (brain MRI negative/spinal cord MRI positive) and 114 (2%) scans showed asymptomatic Gd + lesions in both the brain and SC (brain MRI positive/SC MRI positive) (Fig. 2).

#### Patient-wise

Out of 830 patients, 519 (62.5%) and 387 (46.6%) patients had at least one scan showing new T2 and Gd + lesions in the brain only, while 268 (32.3%) and 217 (26.1%) patients had at least one scan showing new T2 and Gd + lesions in the spinal cord only. Asymptomatic Gd + lesions occurred in 41.3% of patients in the brain only and in 16.1% of patients in the spinal cord only.

### Association between brain and spinal cord Gd + lesions and concomitant relapses

The presence of Gd + lesions in the spinal cord alone (OR = 4.1, 95% CI 2.4–7.1, *p* < 0.001), and concurrently in the brain and spinal cord (OR = 4.9, 95% CI 4.6–9.1, *p* < 0.001), were associated with an increased risk of concomitant relapses when compared to the presence of Gd + lesions in the brain a lone (Table 3).

**Table 3:** Logistic regression model for the risk of concomitant relapse by patterns of Gd + lesions on MRI scans (*n* = 5701)

	Relapses			Nagelkerke pseudo-*R*^2^		
	No *n* = 5127	Yes *n* = 527		0.29	0.19	0.11
Brain negative/spinal cord negative *n* = 4521	4314 (95.4%)	207 (4.6%)	OR 95% CIs *p*-value	1.0 [REF]	–	–
Brain positive/spinal cord negative *n* = 657	540 (82.2%)	117 (17.8%)	OR 95% CIs *p*-value	2.9 1.8 to 4.5 ** < 0.001**	1.0 [REF]	–
Brain negative/spinal cord positive *n* = 286	159 (55.6%)	127 (44.4%)	OR 95% CIs *p*-value	10.7 6.5 to 17.4 ** < 0.001**	4.1 2.4 to 7.1 ** < 0.001**	1.0 [REF]
Brain positive/spinal cord positive *n* = 237	114 (48.1%)	123 (51.9%)	OR 95% CIs *p*-value	13.4 7.5 to 23.9 ** < 0.001**	4.9 4.6 to 9.1 ** < 0.001**	1.4 0.7 to 2.5 0.34

Between group post hoc comparisons were carried out versus the first reference category (brain MRI negative/spinal cord MRI negative) [REF] and then in a repeated way versus the previous category

Model weighted for the no. of scans per patient and adjusted by sex, age, disease duration, EDSS score, DMT taken and the presence of a concomitant relapse at the time of each MRI scan

Bold values denote statistical significance at the *p* ≤ 0.05 level

### Association between brain and spinal cord new T2 lesions and disability worsening

Disability accrual occurred in 341 patients after a median time of 1.5 (range < 1–15) years. When the association between MRI patterns and disability worsening was investigated, only the presence of new T2 lesions in both the brain and spinal cord was associated with an increased risk of disability worsening when compared to the presence of new T2 lesions in the brain alone (hazard ratio [HR] = 1.4, 95% CI = 1–2.1, *p* = 0.05) (Table 4). No association between the risk of disability accrual and MRI patterns based on symptomatic or asymptomatic Gd + lesions was found.

**Table 4:** Cox regression model for the risk of disability accrual according to patterns of new T2 lesions on MRI scans (*n* = 4872)

	Disability accrual			Generalized pseudo-*R*^2^		
	No *n* = 4,374	Yes *n* = 498		0.14	0.12	0.08
Brain negative/spinal cord negative *n* = 3285	2965 (90.3%)	320 (9.7%)	HR 95% CIs *p*-value	1.0 [REF]	–	–
Brain positive/spinal cord negative *n* = 909	820 (90.2%)	89 (9.8%)	HR 95% CIs *p*-value	1.0 0.8 to 1.3 0.90	1.0 [REF]	–
Brain negative/spinal cord positive *n* = 301	265 (88.0%)	36 (12.0%)	HR 95% CIs *p*-value	1.2 0.9 to 1.8 0.24	1.3 0.9 to 1.9 0.22	1.0 [REF]
Brain positive/spinal cord positive *n* = 377	324 (85.9%)	53 (14.1%)	HR 95% CIs *p*-value	1.5 1.1 to 2.0 **0.02**	1.4 1.0 to 2.1 **0.05**	1.2 0.7 to 2.0 0.42

Between group post hoc comparisons were carried out versus the first reference category (brain MRI negative/spinal cord MRI negative) [REF] and then in a repeated way versus the previous category

Model weighted for the no. of scans per patient and adjusted by sex, age, disease duration, EDSS score, DMT taken and the presence of a concomitant relapse at the time of each MRI scan

Bold values denote statistical significance at the *p* ≤ 0.05 level

## Discussion

We investigated the frequency of MRI activity in the spinal cord, alone and in combination with brain activity, and their association with relapses and disability accrual, in a large, single-center cohort of treated patients with MS, mostly with relapsing disease course, longitudinally followed as per clinical practice.

We found that about one third of patients showed new T2 lesions exclusively within the spinal cord, and 16.1% of patients showed asymptomatic Gd + lesions exclusively in the spinal cord.

Our findings extend the results of a previous study, which reported asymptomatic spinal cord lesions in one-fourth of clinically stable MS patients and spinal cord lesion activity alone in 10% of clinically stable MS patients [18–20]. The majority of our patients was on treatment, and this may explain the high percentage of scans which showed no new T2 lesions and no Gd activity in both the brain and spinal cord over the entire follow-up (68.6% and 79.3%, respectively). In our study, we found that asymptomatic Gd + lesions were seen exclusively in the spinal cord in 2.8% of the scans, and this is a lower percentage than that previously reported (between 9.8 and 12%) [20, 21]. This difference may be explained by possible sampling biases of previous studies, since spinal cord MRI was requested when clinically indicated, leading to a possible over-representation of scans (and patients) with spinal cord lesions. In addition, there were differences in the clinical phenotypes included in the analysis and in length of the follow-up between studies.

Spinal cord lesions are thought to be more likely symptomatic and leave residual neurological impairment, due to poor compensatory capacity of the spinal cord, than brain lesions [22]. In line with these findings, in our population new activity within the spinal cord (presence of Gd + lesions) was associated to higher risk of experiencing a concomitant clinical relapse in comparison with new brain activity. However, 16.1% of enrolled patients still presented asymptomatic, isolated spinal cord activity (Gd + lesions). The opportunity for a therapeutic re-evaluation in patients with asymptomatic, isolated spinal Gd + lesions would have been missed limiting the MRI protocol to brain monitoring alone. Indeed, the demonstration of the occurrence of inflammatory activity at spinal cord level independently from brain lesions might have an impact on treatment decisions, as it allows the identification of a proportion of patients who would otherwise be considered stable [10, 23]. In general, spinal cord lesions are important predictors of switching therapy, as demonstrated in in a survey on 3025 patients newly diagnosed RRMS patients from 24 Italian centers [8].

The predictive value of spinal cord involvement has been described in subjects with early disease. In patients with radiologically isolated syndrome, the presence of spinal cord lesions was seen in 64% of patients who later developed CIS or MS [24]and in 100% of patients who later were diagnosed as primary progressive MS [25]. In patients with CIS, the presence and the number of spinal cord lesions were associated with increased risk of clinical conversion to MS (OR: 14.4; 95% confidence interval: 2.6–80.0) regardless of demographics, clinical features and brain MRI [26, 27].

We found that the presence of new T2 lesions at both brain and spinal cord levels was associated with an increased risk of disability accrual, compared to new T2 lesions in brain only. Conversely, the presence of new Gd + (symptomatic and asymptomatic) lesions was not associated with the risk of disability worsening. This is in line with several report in the literature [28, 29], showing how Gd + lesions do not represent robust predictor of future disability worsening. This might be explained by the different pathological processes undergoing during the first stage of demyelinating lesions compared with chronic inactive ones [30]. Moreover, the role of slowly expanding lesions that are not characterized by disruption of blood brain barrier, as predictors of neurological impairment has shed light on chronic compartmentalized inflammation as one of the possible mechanisms contributing to disability progression [31]. To date, this matter has not been explored at spinal cord level. Indeed, spinal cord T2 lesions detected by MRI reflect focal demyelination and reduced axonal density [32] that is more often seen in patients with progressive disease [33] and correlate with physical impairment [34]. In a report that quantified cervical cord lesion load on axial images with high in-plane resolution MRI, patients with secondary progressive MS not only had a higher spinal cord lesion burden compared with RRMS patients, but this correlated with physical disability, independently from the influence of cord or brain atrophy [35]. Similar results were recently highlighted in a very large sample of patients with RRMS [5]. These findings confirm that local spinal cord damage plays an important role in disability progression, as further supported by a recent work which identified the presence of simultaneous brain and spinal cord atrophy as the strongest correlate of progression over the short term (3.7 years) in a cohort of RRMS patients [36]. Moreover, spinal cord imaging can nowadays be routinely and easily acquired on clinical scanners, given the technical advances that have improved spinal cord acquisition (i.e., 20 min acquisition time) and imaging with enhanced image resolution [37].

Our work is not free of limitations. First, this is a retrospective study that could have suffered from methodological limitation (e.g., the extent of symptoms may not have been completely recorded; charting could have been incomplete; transcription of report not correct) and we could not control for other events that might have occurred during the follow-up. However, we attempted to minimize these concerns double checking all the clinical and MRI data previously collected in the registry. Although we based our evaluations on a radiological report rather than a visual inspection and counting of new T2/Gd + lesions, we systematically perform a verification of the radiological report before entering data in the registry. Moreover, our results are based on lesion counts rather than lesion volumes in the spinal cord and there is a growing evidences demonstrating that lesion volume evaluation might be superior to lesion count as an explanation for damage and clinical disability in MS [38]. Furthermore, the length of follow-up was variable across patients, and further events (such as relapses, disability worsening, and conversion to secondary progressive MS) could have occurred during a longer observation. Nevertheless, we tried to control for the follow-up variability by taking into account the number of sessions for each patient in the statistical analysis. Finally, clinical stability was defined by the absence of relapses and stable EDSS scores, but these measures might be not sufficiently sensitive to reflect subtle changes in neurological function at time of spinal lesions appearance.

Our data, derived from a long-lasting experience in a large cohort of MS patients, demonstrated that spinal cord activity (new T2 lesions) occurs independently of brain activity in 32.3% of patients with MS, that they are associated with a higher risk of concomitant relapses and, together with brain MRI activity, are associated with disability progression. Even though asymptomatic Gd + spinal cord lesions alone developed in a small percentage of scans (2.8%), they still occurred in 16.1% of patients during the follow-up. Overall, our data suggest that a routine MRI protocol, including both brain and spinal cord MRI, may help to detect a proportion of patients who would otherwise be considered stable, while possibly requiring a revision of their treatment plan, with relevant implications on long-term disability outcomes. In addition, the presence of new spinal cord lesions, either alone or in association with new brain lesions, increases the risk of both relapses and disability worsening when compared to the presence of new brain lesions alone. Therefore, monitoring MS with spinal cord MRI may allow a more accurate risk stratification and individual treatment optimization.

## Data Availability

The raw data supporting the conclusions of this manuscript will be made available by the authors, without undue reservation, to any qualified researcher.
